# *In vivo* models of *Escherichia coli* infection in poultry

**DOI:** 10.1186/s13028-022-00652-z

**Published:** 2022-12-02

**Authors:** Sofie Kromann, Henrik Elvang Jensen

**Affiliations:** 1 Department of Veterinary and Animal Sciences, Faculty of Health and Medical Sciences, University of Copenhagen, Ridebanevej 3, 1870 Frederiksberg C, Denmark; 2 DanHatch Denmark A/S, Rugerivej 26, 9760 Vrå, Denmark

**Keywords:** Animal models, APEC, Colibacillosis, *E. coli*, Experimental models, Infection models

## Abstract

*Escherichia coli* represents a significant challenge to the poultry industry due to compromised animal welfare, vast productivity losses, elevated mortality, and increased use of antimicrobial compounds. Therefore, effective preventive strategies and insight into the pathogenesis and disease mechanisms of colibacillosis are essential to secure a healthy poultry production. Consequently, discriminative *in vivo* models of colibacillosis are prerequisite tools for evaluating e.g., preventive measures, exploring novel treatments and understanding disease development. Numerous models of colibacillosis are applied for experimental studies in poultry. Yet, few studies provide a proper characterisation of the model enabling other authors to reproduce experiments or use the model in general. The present paper provides a literature review on avian *in vivo* models of primary colibacillosis.

## Background

In poultry, infection with avian pathogenic *Escherichia coli* (APEC) constitutes a significant health challenge compromising both animal welfare, productivity and results in the usage of antimicrobial drugs [1, 2].

As one of the commonest diseases in the most abundant domestic livestock species worldwide [3], colibacillosis is a major contributor to vast economic losses and widespread animal suffering. Thus, means to control colibacillosis, e.g., effective vaccines, are highly warranted, and in-depth insight into pathogenesis and disease mechanisms is vital.

To ensure effective control, whether therapeutic or preventive, discriminative animal models are essential. Likewise, there is a need for valid *in vivo* models in the quest to study the mechanisms behind the disease [4].

When evaluating *in vivo* models the concepts of validity must be considered. In models used to assess preventive strategies or therapeutic effectiveness, it is essential that the model holds proper predictive validity—i.e., are the results obtained using the model similar to the outcome within the spontaneous infections? In other words, how well does the model predict what would truly happen within the natural host of the disease [4, 5]. Failure of a model to exhibit proper predictive validity can result in a false sense of security if, e.g., a vaccine is ineffective under field conditions, treatment fails, or it could, conversely, lead to faulty abandonment of, e.g., preventive strategies, which could have hindered animal suffering and economic losses. Construct validity is particularly important when investigating disease development as this concept applies to the similarity between the mechanisms resulting in disease within the natural host and the model [4]. Thus, are the pathogenesis and mechanisms of disease within the model similar to the naturally occurring condition? Or could it be, e.g., unnecessarily invasive and lack important steps of disease development. Face validity concerns the model’s ability to mirror the actual condition. For example, if the clinical signs and pathomorphology of lesions are similar to those of spontaneous disease [6].

*In vivo* models exhibiting high validity on these essential concepts exemplifies a discriminative animal model. A discriminative infection model would, therefore, mimic the natural route of infection, pathogenic agent, disease progression, clinical signs, gross and histopathological changes, and the immunocompetence should be equal to that of the natural host succumbing to disease [6].

Another vital concept in animal studies is the appropriate usage of a proper study design, e.g., randomisation, blinding and adequate controls [7]. Likewise, proper reporting of methods and results is essential. The methods should be described in sufficient detail, allowing others to evaluate and use the model, whilst adequate reporting on outcomes and results enables researchers to assess whether the model fits their study.

In the present review, the primary aim was to examine the available literature, describing the development or characterisation of *in vivo* models of colibacillosis in poultry as their main purpose. This was done to establish an overview of inoculation methods, inoculum characteristics, animal characteristics, housing conditions and the methods applied to assess the outcome of the study. Evaluation of the experimental study design constituted a secondary aim, seeking to elucidate the use of *lege artis* principles, including randomisation and blinding, in* in vivo* studies conducted in poultry.

## Search strategy

A systematic review was conducted through a literature search of the electronic databases PubMed [8] and Web of Science [9], with “All databases” set as default in the latter. The search terms were as illustrated in Table 1, and only peer-reviewed papers eliciting original research utilising avian animal models of *E. coli* infections were included in the current review. Inclusion criteria were studies stating the development and/or characterisation of an *in vivo* model of colibacillosis as a main study scope, purpose or aim. Exclusion criteria were as follows: all non-original research articles (e.g., meeting or conference abstract), models without application of viable *E. coli* bacteria (e.g., lipopolysaccharides instead of live bacteria) and models applying dual/co-infections with other pathogens or pharmaceutical immunosuppression. In addition, studies utilising only avian embryos were excluded as well as colonisation- and, e.g., transmission studies not aiming to cause or evaluate infection. Publications written in other languages than English were also excluded. Abstracts of all the papers identified through the two databases were thoroughly assessed for their relevance to decide upon either inclusion or exclusion. The included papers were reviewed in detail, and systematic registrations were made, including, but not limited to, the use of controls, randomisation, and blinding, group sizes, animal information (e.g., breed gender, age), methods utilised in the assessment of the model and the use of statistics for evaluation.

**Table 1:** Keywords and combinations

“*E. coli*” OR “*Escherichia coli*” OR colibacillosis
AND
avian OR poultry OR fowl OR bird* OR chicken*
AND
“animal model*” OR “avian model*” OR “poultry model*” OR “turkey model*”
“broiler model*” OR “experimental model*” OR “infection model*” OR “model* of infection”

## Review

Abstracts from PubMed (n = 561) and Web of Science (n = 583) were identified through the search terms and subsequently examined. Of these, a total of 14 papers met the inclusion criteria without succumbing to any of the criteria of exclusion. A single study not explicitly stating model development as a main goal was included due to the application of a route of inoculation (intra-navel) not described by the other studies [10]. Although the authors have made considerable efforts to identify relevant publications, some might have been missed due to, e.g., unclear study aims, not being obtainable online or identified by the included search terms.

Amongst the described models, the majority were conducted in the species *Gallus gallus domesticus* of both laying and meat-production types and of various ages (Table 2). The reports on the breed or line of animals, as well as research animal provider, varied, whereas the age of the birds and group sizes were readily provided. Other information related to the animals, such as housing conditions, temperature within the facilities and the feed provided, was often sparse (Table 2).

**Table 2:** Study design and animals

Study aim/purpose/scope etc.	Species, race	Research animal provider	Age	Gender	Group sizes (n)	Housing	Feed	Light/dark cycle etc	Randomised group allocation (yes/no)	Additional information (SPF, immunosuppression, analgesia etc.)	References
Establishes a model of yolk sac infection (not a main aim)	Chicks	Sunrise farms	12 h	NA	NA	NA	*Ad libitum* chicken starter ration	NA	No	Derived from SPF eggs	[10]
Establish a model of differential resistance towards APEC	Inbred lines: 7_2_, 15I and C.B12. Novogen brown layers Repeat study: 7_2_, 15I	National Avian Research Facility, University of Edinburgh	2 weeks	NA	10/each line and each dose Repeat study: 18	NA	*Ad libitum*	NA	No	All inbred lines were hatched and housed under SPF conditions Novogen Brown layers were housed under SPF conditions	[12]
Describe a model useful for studying ExPEC infections and a chicken lung colonization model	White leghorn	Lohman Tierzucht GmbH	5 weeks	NA	Approx. 15^a^ Depends on experiment	NA	*Ad libitum*	NA	No	SPF	[11]
Develop a reproducible infection model ideally mimicking natural infection	White leghorn layers	NA	56 weeks	Female	5	NA	*Ad libitum*	NA	No	Two initial pilot studies are described in detail in the paper	[13]
Develop a cellulitis model with numerous manifestations of colibacillosis	Broilers	Department of Animal and Poultry Science, University of Saskatchewan, Canada	3 weeks old	NA	Dose titration: 13 (12) Bacteraemia: 10 (9)	Isolation rooms	Commercial broiler ration *ad libitum*	12 h lighting period	Yes	20–22 °C in the facilities Non-recirculated air with a rate of 8–10 changes/h	[14]
Describe lesions of colibacillosis in a turkey (stress) model	Slow growing turkey line with high egg yield, a line selected for high growth, and a commercial turkey line	Slow- and fast-growing lines: Ohio Agricultural Research and Development Center, Wooster, Ohio Commercial line: commercial turkey hatchery	14 weeks at challenged	Mixed sex	7–10 in challenge groups, 5–6 in control groups	Floor pens with pine shavings	*Ad libitum* standard corn and soybean turkey ration	23 h day, 1 h night	No	Obtained at hatch Sexed upon necropsy	[15]
To develop and describe an aerogenous model of avian colibacillosis in adult broiler breeders	Ross 308	SweHatch, Sweden	29 weeks upon arrival	Female	20	8.64 m^2^ coops/group with wood shavings, turf dust-baths, straw, hay, shelves, and perches as enrichment	155 g/hen/day commercial wholefood for egg-laying hens	12 h/12 h with 30-min dim-phases	Yes	Treated with buprenorphine upon indication 7 days of acclimatation Housing temperatures provided	[5]
To reproduce peritonitis syndrome with *E. coli* in layers^b^	Brown layers	Commercial	23–33 weeks depending on study	Female	7 or 14 depending on study	Negative-pressure high-efficiency particulate air isolators of 1.32 m^3^. Housed separately	*Ad libitum* commercial layer diet	16 h of light	No	Isolator temperature, relative humidity, and ventilation rate 7 days of acclimatation Euthanasia by carbon dioxide/O_2_ mixture and bleed from *vena jugularis* Provides vast detail on inoculation methods	[16]
Describe a modified model of cellulitis under realistic conditions^b^	Broilers	Commercial hatchery	28 days (assigned to treatment groups)	NA	250^b^	Floor pens with pine shavings	*Ad libitum*	NA	Yes	Exp. 1: total n = 1500 Exp. 2: total n = 1600 Euthanasia was performed by cervical dislocation Moribund or disabled birds were euthanised immediately	[22]
Validation of an *E. coli* model in Japanese Quail	Japanese quail	Fircrest Farms	9 weeks old at arrival	NA	10	Floor pens	Turkey grower feed (non-medicated) *ad libitum*	NA	Yes	Controlled ventilation within facilities and a temperature kept at 20–21 °C 7 days of acclimatation Euthanasia by isoflurane inhalation followed by cervical dislocation	[17]
Refine challenge procedures and develop a reproducible model of cellulitis further	Broilers	Commercial hatchery	39 days	NA	Exp.1: 10^a^ Exp.2: 85 altogether	Floor pens with new litter	*Ad libitum*	NA	Exp.1: Yes	Moribund and surviving animals were euthanised by carbon dioxide asphyxiation	[18]
Describe a realistic model of cellulitis	Broilers	Commercial hatchery	28 days	NA	Approxima-tely 133/group	Floor pens	*Ad libitum*	NA	Yes	Moribund and surviving animals were euthanised by carbon dioxide asphyxiation or cervical dislocation	[21]
Develop a model in 3–4-week-old chickens of colibacillosis with an exposure simulating the natural route	Commercial chickens	Commercial hatcheries	Arrives as day old chicks. Age at the day of exposure depends on experiment	NA	Dependent on experiment. The study includes numerous experiments	Initially battery cages. Transferred to Horsfall units on the day of inoculation	*Ad libitum*	NA	Yes	Schematic drawing of cone-chamber	[20]
To develop a surgical model based on oviduct inoculation^c^	Commercial white layers	NA	48 weeks	Female	5	Housed on the floor	NA	NA	No	All birds received buprenorphine prior to surgery	[19]

APEC, avian pathogenic *E*. *coli*; exp., experiment; ExPEC, extraintestinal pathogenic *E. coli*; h, hour; NA, not available; SPF, specific pathogen free

^a^Multiple experiments were conducted with the n varying slightly

^b^Not stated directly in the paper

^c^The study had several aims

A wide span of inoculation routes was exploited to establish colibacillosis (Table 3). Those considered invasive were based on direct inoculation into the vagina, uterus, oviduct, trachea, peritoneum, air sac, or subcutis (Table 3) [10–19]. Among these, intratracheal (IT), oviduct, intraperitoneal (IP), intra-air sac (IAS) and subcutaneous (SC) inoculation would be considered most invasive, and thus, those having the lowest construct validity, as they bypass important steps of the initial pathogenesis and natural defence mechanisms. Yet, utilising these inoculation routes might still provide valuable results for some studies. Contrary to invasive methods of inoculation, a number of models sought to mimic the natural infection closely by applying, e.g., aerosols [5, 16, 20]. Also, several studies reported models of cellulitis, which were based on superficial scratching of the skin followed by housing on litter inoculated with *E. coli* [21, 22]. Though the pathogenesis of cellulitis in broilers is not completely clear, invasion through a compromised skin barrier is definitely likely to be a common route of infection [22, 23], however, it could also be a result of *E. coli* septicaemia. The models listed in this review were capable of reproducing characteristic lesions through skin scratches [21, 22]. Coliform cellulitis is an important and high-cost manifestation in broilers due to condemnations, and, therefore, it is not surprising that multiple models have been developed [14, 18, 21].

**Table 3:** Bacterial strain and inoculum

Bacterial strain information	Origin of the strain	Growth state	CFU (dose)	Volume (mL)	CFU/mL	CFU confirmation	Method of *E. coli* inoculation	Vehicle/broth	Positive control	Negative control	Time of euthanasia	References
EC317, O2:K1	Isolated from a turkey with septicaemia	NA	Experiment 1: 1 × 10^4^, 1 × 10^5^ Experiment 2: 25, 250, 2500	100 µL	Dose dependent	NA	Intra-naval performed using a 27G needle and a 1 mL syringe	PBS	Not applicable	No	NA/reach of humane endpoint	[10]
APEC O1 (O1:K1:H7)	Supplied by Prof Lisa Nolan, Iowa State University, USA	Stationary phase	Low dose: 2 × 10^5^ High dose: 1.9 × 10^7^ Repeat study: low, intermediate and high dose	100 µL	Dose dependent	Serial dilutions	100 µl PBS containing bacteria into the right caudal thoracic air sac	Luria Bertani broth, centrifuged and resuspended in PBS	Not applicable	In repeat study a sterile PBS control was included (n = 3)	2 dpi and 7 dpi Repeat study: 14 h, 3 dpi, 7 dpi	[12]
IMT5155, O2:K1:H5	Outbreak isolate from the internal organs of a laying hen	Presumed exponential phase. Overnight culture diluted and grown at the day of inoculation, centrifuged and resuspended in PBS	1 × 10^9^ Depends on experiment	0.5 mL	10^9^	Serial dilutions	IT	Luria Bertani broth, centrifuged and resuspended in PBS	Not applicable	Intestinal *E. coli* isolate (IMT11327) and PBS	24 and 48 h dpi	[11]
APECO78	Obtained from the ovary of a hen that died from egg yolk peritonitis	Grown until an optic density of 0.6–0.8	1 × 10^9^	100 µL stated in pilot study	NA	NA	IU bacterial inoculation (1 mL syringe introduced through the vagina into to the uterus) and 2–3 mL egg yolk IP	Luria Bertani broth, centrifuged and washed in PBS and resuspended in egg yolk	Not applicable	Egg yolk IP and IU (Additionally, *E. coli* K12 during pilot studies)	7 dpi	[13]
O78 (nonhemolytic, aerobactin positive, serum resistant ad K1 negative)	Field isolate from broiler with cellulitis, pericarditis and airsacculitis	Grown for 12 h	Ranging from 1 × 10^5^ to 1 × 10^9^ (Bacteraemia induction 1 × 10^7^)	1 mL	Ranging from 1 × 10^5^ to 1 × 10^9^	Serial dilutions	SC injection into the left caudal abdominal region	Grown in BHI, washed and resuspended in saline	Not applicable	Saline	Dose titration: 7 dpi Bacteraemia: 6 h and 1, 3, 5, 7 and 14 dpi	[14]
*E. coli* O2	Isolated from a chicken with septicaemia	Grown for 2.5 h	Approx. 1 × 10^3.7–4^	NA	NA	Serial dilutions	Inoculated into the left cranial thoracic air sac. At 8 dpi the birds were loaded onto a transportation vehicle, driven for 3 h and subsequently kept in the truck an additional 9 h. Temperature: 18–21 °C	Tryptose phosphate broth	Dexamethasone receiving groups	Yes, non-stressed and not receiving *E. coli*	2 weeks dpi	[15]
ST117 E44 (accession number LXWV00000000.1)	Clinical case of colibacillosis	Exponential phase (4 h growth at the final preparation step)	1.9 × 10^7^^a,b^	Not applicable	1.9 × 10^7a,b^	Serial dilutions	Inhalation of aerosolised inoculum while placed in a specialised chamber Aerosols were produced utilising Omron Ultrasonic Nebulizer NE-U17 (IT: buttoned cannula)	Lysogeny broth	Yes, IT	Sterile lysogeny broth (aerosolised and IT)	2, 4, 5 and 7 dpi	[5]
chicken/NL/Dev/SP01404Cou/05	Hen with peritonitis from a flock with high mortality	17 h growth prior to inoculation	1 × 10^7.6–9.1^ Aerosols^b^: 1 × 10^5.1–5.7^ and 1 × 10^6.2^	1 mL (Aerosols: 100 mL diluted culture)	1 × 10^7.6–9.1^	Bacterial counts	Numerous including IV (*vena ulnaris*, 23G needle), IP (23G needle), IT (knobbed, curved cannula), IVAG (knobbed, curved cannula), aerosol (1–4 exposures, spray head coupled to air compressor) and oral (knobbed, curved cannula) Some received 25 mL sterile egg yolk IP prior to *E. coli* inoculation (50 mL syringe, 18G needle)	Glucose broth (Aerosols: buffered peptone water)	Not applicable	IP or IT buffered peptone water	2–4 weeks after inoculation	[16]
EC-AR1, EC-CA1, EC-ALN1, EC-CA2, EC-ALN2, EC-AR2, EC-ALS1	US cases of cellulitis in commercial broilers	Refers to [18]	Refers to [18]	Not applicable	1 × 10^8^	Refers to [18]	Exp. 1: Scratches (2–3 cm and two on each bird paralleling the keel (to distinguish these from naturally occurring scratches), made with 18G needle) and placed on litter sprayed with 200 mL sterile saline or *E. coli* (10^8^ CFU/mL) Exp. 2: Scratching as in exp. 1 and then placed on old litter from a previous cellulitis experiment	Refers to [19]	No/not applicable	Scratches and un-scratched birds on litter sprayed with 85% sterile saline	7 dpi	[22]
EC317	Turkey with cellulitis, airsacculitis and pericarditis	13 h of growth in BHI	Dose ranged from approx. 2 × 10^3.3^ to 2 × 10^7c^	200 µL	Ranged between 1 × 10^4^ and 1 × 10^8^	Plate count method	SC into breast	BHI	Not applicable	BHI	21 dpi Earlier if severe clinical signs	[17]
Cellulitis isolate	Cellulitis lesion in a broiler from Arkansas	Stationary (overnight culture)	Exp.1: 1 × 10^8^ (main culture diluted 1:10) Exp.2: overnight culture	1 mL	1 × 10^9^	Serial dilutions	SC with a 1.5-inch, 20-Gauge needle either on dorsum, ventrum or the inguinal area (specified in the article) Swab onto area with plucked feather follicles (2–3 feathers plucked) Swab onto area with scratches (sterile scalpel used to induce scratches)	Exp.1: Sterile physiological saline Exp.2: Brain–heart infusion broth	Not applicable	Exp.1: 10 uninoculated	Exp.1: 7 dpi Exp.2: 6 h interval until 72 h thereafter 8 were killed weekly	[18]
EC-AR1, EC-ALN1, ECALS1, EC-CA1	Refers to [18]	Refers to [18]	Not applicable	Not applicable	1 × 10^8^	Refers to [18]	Scratches with a length of 2–3 cm were induced with a 1.5-inch, 18-Gauge needle on the ventral surface paralleling the keel and subsequently placed on litter with 200 mL sterile saline/2.5 m^2^ or containing 10^8^ CFU/mL	Sterile saline sprays on litter	No/not applicable	Scratched birds on un-inoculated litter Non-scratched birds	7 dpi	[21]
EC99 (O78), EC317 (O2)	Colisepticaemia in chicken and in turkey	16 h growth	Not applicable/Aerosol	Not applicable (100 mL approx. was aerosolized)	1 × 10^9^	Yes	Aerosols made with an Ultrasonic nebulizer. Administered in Horsfall unit (0.155 m^3^) or cone-shaped aerosol chamber (0.123 m^3^) depending on experiment	Grown in tryptic soy broth, washed 3× in PBS and resuspended in PBS	Not applicable	In one experiment, and PBS control was included	Depending on study 6 dpi (primarily) and 21 dpi	[20]
*E. coli* strain C10821, ST117, O111:K_:H4	Avian *E. coli* outbreak	Stationary (overnight culture)	8.6 × 10^6^	0.1 mL	8.6 × 10^7^	Serial dilutions	Deposition directly into the oviduct during surgical procedure under general anaesthesia	Luria broth	Not applicable	Sterile broth	48 h dpi	[19]

BHI, brain heart infusion; CFU, colony forming units; dpi, days post-infection; exp., experiment; h, hour; IP, intraperitoneal; IT, intratracheal; IU, intrauterine; IVAG, intravaginal; NA, not available; PBS: phosphate-buffered saline; SC, subcutaneous

^a^Average as the study was reproduced a second time on a separate day

^b^Dose estimate calculated based on inhaled air volume and respiratory frequency

^c^Calculated by the authors of this review

The intravaginal (IVAG) and intrauterine (IU) models reproduced peritonitis variably [13, 16], and one study reported infection highly aided by concurrent IP administration of sterile egg yolk [13]. A study, depositing *E. coli* directly into the oviduct during a surgical procedure, reported peritonitis but not signs of salpingitis though cultures from the oviduct yielded *E. coli* growth [19]. An IAS model described subjected the birds to transportation stress following inoculation to aid induction of disease [15]. In this study, they reported an increased susceptibility to infection in males and fast-growing lines of turkey, hence underlining the effect of animal characteristics and, thus, the necessity to properly state such information within publications.

Reports on the inoculum were generally comprehensive, with details on isolate origin, dose, vehicle used etc. (Table 3). However, the importance of information on dose (colony forming units (CFU) received by each animal), and the volume the bacteria is administered within, should still be emphasised, as it has previously been reported that, e.g., the concentration can impact the severity of disease [24] or that the sheer volume might act as an irritant *per se* [5].

Evaluation of the disease outcome by histopathology was not particularly common among the identified studies, whereas gross pathology was readily reported (Table 4). Also, numerous studies provided a detailed scoring or recording scheme for gross pathology [11–13, 15, 19, 20]. Likewise, microbiology and statistics were widely applied to assess the models (Table 4).

**Table 4:** Methods of assessment

Microbiological assessment	Gross pathological assessment	Histopathological assessment	Statistical assessment	Other assessments^a^	Randomisation	Blinding	References
Liver, spleen, and yolk sac was cultured	No. Evaluated in subsequent trial investigating immune stimulants	No	No. Used in subsequent trial investigating immune stimulants	No	No	No	[10]
Yes. Bacterial load in lung, blood (CFU/mL), and liver Defined anatomical parts of the organs were used for determination of CFU/g	Yes. Lesions of colibacillosis according to a scoring scheme	No	Yes	Clinical signs (defined signs for mild and moderate)	No	No	[12]
Yes. Tissue samples from lung, kidney, liver, heart, spleen and brain was weighed, homogenised and tenfold dilutions were made	Yes. According to a scoring scheme	Yes	Yes	Multiplex PCR to determine the presence of APEC associated virulence genes of the non-pathogenic strain (IMT11327) Clinical scoring according to a grading system	No	No	[11]
1 g of liver, heart, peritoneum, ovary and oviduct were homogenised in BHI with subsequent tenfold dilutions	Yes. According to a scoring scheme	Yes	Yes	Clinical signs according to a scoring scheme	No	No	[13]
Swabs (cellulitis, pericardium, air sac) onto blood agar and MacConkey Birds found dead: spleen was swabbed in addition Bacteraemia induction: peripheral blood at 6 h and 1, 3, 5, 7 and 14 dpi	Yes	No	Yes	Weight prior to challenge and necropsy	No	No	[14]
Transport swabs were used for sampling of liver, air sac, knee synovial membrane, and all lesions comparable with the turkey osteomyelitis complex	Yes. According to a scheme	No	Yes	Weighed	No	No	[15]
Yes. Swab (right thoracic air sac, liver, spleen, peritoneum, infundibulum and magnum of salpinx, femoral bone marrow, trachea and the left (homogenised) lung	Yes. According to a systematic scheme	Yes	Yes	An air sampler (AeroCollect^®^) was used for CFU measurements within the chamber during exposure Organ/BW-ratio of lung, liver, and spleen	Yes. Stated for gross pathology assessment	Yes. Stated for gross pathology assessment	[5]
Yes. Bone marrow of femur	Yes	No	Yes	Air samples immediately following termination of aerosolization	No	No	[16]
No	Yes	No	Yes	No	No	No	[22]
Swabs inoculated into BHI and subsequently plated onto MacConkey	Yes	No	Yes	BW prior to inoculation and at the time of death Blood samples for bacteriaemia assessment	No	No	[17]
No	Yes	No	No	No	No	No	[18]
No	Yes	No	No	No	No	No	[21]
Yes. Air sac, pericardial sac, and liver (direct streak)	Yes. According to a scoring scheme	No	Yes	No	No	No	[20]
Swabs from the peritoneum, ovary, and the infundibulum, isthmus and uterus of the oviduct with semiquantitative scoring of growth	Yes	Not on animals used to establish the model	Yes	Not on the birds used to establish the model	No	No	[19]

APEC, avian pathogenic *E. coli*; BHI, brain heart infusion; BW, body weight; CFU, colony forming units; H&E, haematoxylin and eosin; IHC, immunohistochemistry; PCR, polymerase chain reaction

^a^Clinical signs are only listed if the study utilises a standardised scheme

A randomised order of animal assessment and blinding was practically absent throughout the examined studies. Particularly, when comparing subjective parameters or subtle changes, blinding is crucial to eliminate bias and should always be prioritised in *in vivo* research studies, whilst randomisation is a key component in controlling variation [7]. Both these elements are also crucial for the 3R principle of “Reduction”, as valid research results are unlikely to be generated without proper control of variation and blinding [7].

As pointed out by Piercy and West in 1976, comparison of results regarding, e.g., pathogenesis and treatments are limited due to a lack of uniformity in experimental techniques highlighting variation in culture media, stage of growth, concentration etc. [25]. In the current review, the authors acknowledge such limitations but wish to emphasise that the lack of sufficient information serves as the main problem as this prohibits studies from being evaluated, compared and/or reproduced. Also, whilst an almost unlimited number of studies apply animal models of colibacillosis, only very few focus on the description and development of these models, thereby failing to provide usable references and share experiences with other authors.

## Conclusions

In the present literature review, an overview of avian models of colibacillosis is given with a comprehensive presentation of details related to the animals, inoculum, inoculation, housing, and model assessment. Numerous portals of infection have been successfully applied to reproduce colibacillosis in poultry with varying degrees of invasiveness. Thus, not all the models would be considered particularly discriminative as essential steps of the natural pathogenesis were often bypassed. Randomisation and blinding during outcome assessment were rarely performed in the studies and should be included as a standard in the future.

## Data Availability

A list of the reviewed literature can be obtained from the corresponding author.
